# PROTOCOL: Adult/child ratio and group size in early childhood education or care to promote the development of children aged 0–5 years: A systematic review

**DOI:** 10.1002/cl2.1079

**Published:** 2020-02-25

**Authors:** Nina T. Dalgaard, Anja Bondebjerg, Rasmus Klokker, Bjørn C. A. Viinholt, Jens Dietrichson

**Affiliations:** ^1^ VIVE—The Danish Center for Social Science Research Copenhagen Denmark

## Abstract

This is the protocol for a Campbell review. The objectives are as follows: To synthesize data from studies to assess the impact of adult/child ratio and group size in ECEC on measures of process characteristics of quality of care and on child outcome measures.

## 1 BACKGROUND

### 1.1 The problem, condition or issue

Worldwide a large number of infants and toddlers are enroled in formal nonparental early childhood education or care. Formal early childhood education or care is defined as professional early childcare or education settings with paid caretakers or teachers as opposed to more informal arrangements such as private babysitters or caretakers consisting of members of the child's extended family. On average across OECD countries around 33% of children aged 0–2 are enroled in early childhood education or care (ECEC), but this ranges from lower than 1% in Turkey to as high as roughly 60% in Belgium and Denmark. For children aged 3–5 the enrolment rates are even higher with an average of 87.2% across the OECD.^1^

Average hours in ECEC also differ across countries. In most OECD countries, children (0–2‐year‐olds) in ECEC attend for an average of somewhere between 25 and 35 hr during a usual week, with the OECD average just under 30 hr per week (see footnote 1). An overall average is not available for 3–5‐year‐olds in the OECD countries, but in Denmark children aged 3–5 years spend an average of 7.5 hr each day kindergarten.^2^ In the developing countries formal childcare is also increasing. In the past 20 years, at least 13 developing countries have instituted compulsory preschool or pre‐primary programmes (Engle et al., 2011), and according to The World Bank roughly half of all children in the relevant age range around the globe were enroled in preschool in 2017.^3^ Thus, with a large number of children spending a substantial number of hours awake every day in nonparental care, it becomes important to examine the impact of the quality of care on the development and well‐being of children.

Quality of care in ECEC may be defined by both structural and process characteristics (Vermeer, van Ijzendoorn, Cárcamo, & Harrison, 2016). Structural characteristics include the adult/child ratio, group size, the formal educational level of staff, years of working experience and in‐service professional development of the caretakers/teachers and the physical child care facilities (Slot, Leseman, Verhagen, & Mulder, 2015). Process characteristics include the caretakers' sensitivity and the quality of the child–caretaker interactions during the day (Schipper, Riksen‐Walraven, & Geurts,  2006). The two aspects of quality of care are associated with each other (NICHD Early Child Care Research Network, 1996). Both structural and process characteristics are associated with positive child outcomes (Auger, Farkas, Burchinal, Duncan, & Vandell, 2014; Burchinal, Cryer, Clifford, & Howes, 2002; Burchinal, Roberts, Nabors, & Bryant, 1996; Howes, Phillips, & Whitebook, 1992; Phillips, Mekos, Scarr, McCartney, & Abbott–Shim, 2000). However, some studies have also failed to find a positive association between a higher adult/child ratio and positive child outcomes (Clarke‐Stewart, Gruber, & Fitzgerald, 1994; Dunn, 1993; Mashburn, Pianta & Hambre, 2008) or have reported mixed results (Howes, 1997).

Structural characteristics of the quality of childcare are readily observable and easier to regulate than process characteristics. However, the specific impact of different aspects of structural characteristics of quality of care on both process characteristics and on child outcomes has yet to be rigorously examined in a systematic review, which is where the present review will contribute. Within the present review, we will examine the effect of two central structural characteristics: adult/child ratio and group size on both process characteristics and on child outcomes.

- https://www.oecd.org/els/soc/PF3_2_Enrolment_childcare_preschool.pdf
- https://www.boerneraadet.dk/media/30309/Miniboernepanel‐Mellem‐hjem‐og‐boernehave.pdf
- https://data.worldbank.org/indicator/SE.PRE.ENRR

### 1.2 The intervention

In this systematic review, we will examine the impact of adult/child ratio and group size on child development and well‐being in formal nonparental early childhood education and care settings. Thus, the intervention is defined as any change to adult/child ratio and/or group size which has been reliably measured within an eligible setting.

Interventions may change the adult/child ratio, the group size, or both simultaneously. That is, to increase the group size while keeping the ratio constant, the number of children needs to increase by exactly the same proportion as the number of adults (e.g., by doubling both the number of children and adults). If an intervention only increases the number of children, the ratio decreases and the group size increases. If the number of adults increases, the adult/child ratio increases while the group size is constant.

In the statistical analyses, we hope to be able to distinguish between interventions that change the adult/child ratio, the group size, or both the ratio and the group size, as well as between high versus low adult/child ratios and between small versus large group sizes.

### 1.3 How the intervention might work

Theoretically, higher adult/child ratios (fewer children per adult) and smaller group sizes are hypothesized to be associated with positive child outcomes. A higher adult/child ratio and a smaller group size are proposed to be associated with an increase in both the extent of and the quality of adult/child interactions during the day. The younger the children are, the more their development and well‐being are proposed to be dependent on adequate, nurturing and stimulating adult/child interactions. Thus, the extent of and the quality of adult/child interactions are by some scholars proposed to be the single most important determinants for the child's development and well‐being within ECEC settings (de Schipper, Riksen‐Walraven, & Guerts, 2006; Christoffersen, Højen‐Sørensen, & Laugesen, 2014; Lamb, 1998; Karoly, 1998; Munton et al., 2002; Vandell & Wolfe, 2000).

Historically, a number of studies suggest that when the adult/child ratio is increased (fewer children per adult) and group sizes are decreased, the number of interactions between each child and an adult increases and the nature of the exchanges becomes more stimulating and nurturing for the child. Thus, caregivers with fewer children in their care have been found to be more sensitive, responsive, warm, nurturing and encouraging towards the children. Furthermore, a higher adult/child ratio has been found to be associated with adults exhibiting more positive and less negative affect, and with adults who provide more varied and developmentally appropriate activities for the children. Previous studies further suggest that when fewer adults are in charge of a larger group of children, the caregivers become more focussed on managing and controlling the children's behaviour. This means that the adults will give more commands and corrections, exert more negative control and spend less time engaged in reciprocal conversations or playful interactions with the children. With lower ratios (fewer adults to children) and larger group sizes, the adults will be more likely to ignore or overhear children's questions and they will spend less time engaged in positive affirmation. Furthermore, early studies suggest that with lower ratios and higher group sizes, children will have more conflicts during free play situations and thus the adults may need to spend more time on acute problem solving (Dawe, 1934; Christoffersen et al., 2014; Gevers Deynoot‐Schaub & Riksen‐Walraven, 2005; Ghazvini & Mullis, 2002; Howes, 1983, 1997; Howes & Rubenstein, 1985; Howes, Smith, & Galinsky, 1995; NICHD Early Child Care Research Network, 1996, 2000; Roudinesco & Appell, 1950; Palmeérus and Hägglund, 1991; Phillipsen, Burchinal, Howes, & Cryer, 1997; Sjølund, 1969; Stallings & Porter, 1980; Volling & Feagans, 1995; Williams & Mattson, 1942). Theoretically, it is also possible that more adults in the same class room will allow for more teacher supervision and support, which may affect the quality of the class room environment positively.

Furthermore, previous studies have also found adult child/ratio and group size to be associated with positive child outcomes such as decreased levels of anxiety, aggressive behaviour and distress, greater social competence and better receptive and expressive language skills (Burchinal et al., 1996; Vernon‐Feagans, Manlove, & Volling, 1996; Volling & Feagans, 1995). Theoretically this may be explained by both the quality and frequency of the adult/child interactions. However, some scholars also suggest that a smaller group size regardless of the adult/child ratio may be beneficial to the group dynamic and may decrease the children's stress levels (Christoffersen *et al.* 2014).

In a large‐scale study in the United States (The National Day Care Study), data from 64 day care centres was collected between 1974 and 1978, and results suggested that for children aged 3–5 years of age, smaller groups had a positive impact on children's development and behaviour, even when the adult/child ratio was the same. Thus, children in smaller groups consisting of 12–14 children with 1–2 adults did better than children in larger groups consisting of 24–28 children with 4 adults on measures of behaviour and school readiness. In the smaller groups, children were more cooperative, less aggressive and had fewer conflicts compared with children in the larger groups, and in the smaller groups there was more positive adult/child interaction than in the larger groups, even when the adult/child ratio was the same. The same findings did not apply to children aged 0–2; for the very young children, both the adult/child ratio and the group size was associated with positive child outcomes (Ruopp, Travers, Glantz and Coelen, 1979; Ruopp, Travers, Glantz, Coelen, and Smith, 1979).

However, findings regarding the impact of adult/child ratio and group size are far from unequivocal, as a number of observational studies have failed to find significant positive associations between adult/child ratio and group size and the expected process quality and child outcomes (Barros & Aguiar, 2010; Fukkink, Gever Deynoot‐Schaub, Helmerhorst, Bollen, & Riksen‐Walraven, 2013; Pessanha, Aguiar, & Bairrao, 2007; Pianta et al., 2005; Vermeer et al., 2008). An example of a study which fails to support the association between group size and adult/child ratio and positive process quality outcomes is Slot et al. (2015). In this study based on a national Dutch cohort study of preschool education and care provisions, child‐to‐teacher ratio and group size did not explain variance in emotional or educational process quality between ECEC classrooms. Similarly, Blau (2000) found a small and statistically insignificant association between group size and child care quality and only a small positive association between adult/child ratio and child care quality in a study based on data from a random sample of day care centres in four different states in the United States.

In summary despite some previous contradictory findings, the adult/child ratio and group size are hypothesized to affect the process characteristics of quality of care, meaning that an increased adult/child ratio and reduced group size are associated with an increase in positive child–caretaker interaction and in caretaker sensitivity, responsiveness, warmth, nurture and encouragement towards the children and with more positive and less negative affect. Furthermore, an increased adult/child ratio and a reduced group size are hypothesized to be associated with positive cognitive, behavioural and socioemotional child outcomes.

### 1.4 Why it is important to do this review

To our knowledge, no systematic review of the effects of both adult/child ratio and group size in ECEC on both the process characteristics of quality of care and on child outcomes has previously been carried out.

Perlman et al. (2017) conducted a systematic review and meta‐analysis of child‐staff ratio in ECEC settings on child outcomes. The purpose of this systematic review was to evaluate the association between child‐staff ratios and children's outcomes. Searches revealed 29 relevant studies, with only three studies eligible for inclusion in the meta‐analysis. These three studies focused exclusively on associations between child/staff ratios and children's receptive language, thus not allowing for broader conclusions regarding child outcomes in other areas, for example, interpersonal skills or child well‐being. Perlman et al. noted that the methodological properties of studies within the ECEC literature may pose a challenge to researchers wishing to conduct statistical meta‐analyses. The methodological issues encountered by Perlman et al. arose from, for example, the operationalization of child‐staff ratios, the child outcome domains measured, the psychometric properties of outcome measures and overall study design, leading the authors to call for more comparative effectiveness research designs, such as prospective cohorts or cluster‐randomized studies (Perlman et al., 2017). It is possible that we may encounter similar methodological challenges in this systematic review.

While the review by Perlman et al. provides important insight, the scope of the present review is broader as we will examine the causal effect of both adult/child ratio and group size and we will include process characteristics of quality of care as outcomes. Furthermore, while the review by Perlman et al. only examined children aged between 30 and 72 months, we will include children in a broader age range. Finally, the present review will include an extensive risk of bias assessment.

Whereas process characteristics of quality of care are difficult to measure and regulate, the structural characteristics are readily observable and easier to regulate. However, improvements in the structural characteristics of the quality of care by more having adults in charge of fewer children in smaller groups are costly. Therefore, it is important to determine the overall and relative efficacy of such improvements in facilitating optimal development and well‐being in children attending ECEC.

## 2 OBJECTIVES

To synthesize data from studies to assess the impact of adult/child ratio and group size in ECEC on measures of process characteristics of quality of care and on child outcome measures.

## 3 METHODS

### 3.1 Criteria for considering studies for this review

#### 3.1.1 Types of studies

In order to summarize what is known about the causal effects of adult/child ratio and group size on process quality characteristics and child outcomes in ECEC settings with children aged 0–5 years, we will include all studies with a well‐defined control group. Thus, the study designs eligible for inclusion are:

1 Controlled trials
  - Randomized controlled trials (RCTs)
  - Quasi‐randomized controlled trial designs (QRCTs). Here participants are allocated by means, which are not expected to influence outcomes, for example, alternate allocation, participant's birth data, case number or alphabetic order.
2 Quasi‐experimental studies (QES), This category refers to both studies, where participants are allocated by other actions controlled by the researcher, or where allocation to the intervention and control group are not controlled by the researcher (e.g., by time differences or policy rules).

To be included, QESs must credibly demonstrate that outcome differences between intervention and control groups are the effect of the intervention and not the result of systematic baseline differences between groups. That is, selection bias should not be driving the results. This assessment is included as part of the risk of bias tool, which we elaborate on in the Risk of bias section.

In order to include all relevant data, we will also include studies using a repeated‐measures experimental design in which the same caregiver and/or children are observed under different conditions within a short time span. In such a design, children and caregivers act as their own control group. As children and caregivers develop their skills over time, single group repeated‐measures designs are prone to confounding intervention effects with naturally occurring child and caregiver development. Therefore, we will only include repeated‐measures designs with time spans where natural skill development is likely to be minimal (i.e., days rather than months).

The aim of the present review is to summarize evidence regarding the causal impact of both adult/child ratio and group size on both process characteristics and on child outcomes, and thus we will exclude studies reporting associations in cohort, cross‐sectional and longitudinal study designs, if they do not include a relevant comparison group.

In order to minimize the risk of bias, we will exclude study designs in which only one unit was assigned to the intervention or control group. That is, there must be at least two units in the intervention group and two units in the control group, otherwise there is a very high risk of confounding treatment effects with “unit” effects (unit would likely be preschool/childcare centre/daycare(r) in our case). Furthermore, we will exclude studies using non‐comparable treatment and control groups, for example, studies that use highly selected groups (as when a study compares at‐risk and not‐at‐risk children).

#### 3.1.2 Types of participants

This review will include studies of children aged 0–5 years who are enroled in some form of formal nonparental ECEC. Formal ECEC is defined as professional settings with paid caretakers or teachers. We will include studies of children with special needs and children considered at risk. We will exclude children living in any kind of residential care arrangements such as foster families or institutions.

#### 3.1.3 Types of interventions

In this systematic review, we will examine the impact of different adult/child ratios and group sizes on child development and well‐being in formal nonparental ECEC settings on child development and well‐being. Thus, eligible interventions are defined as any adult/child ratio and/or group size which has been reliably measured within an eligible setting.

In order to be eligible for inclusion, studies must report either adult/child ratio and/or group size. In measuring these variables, we will accept studies using both direct observation and register‐based data in which the adult/child ratio is derived from information regarding the number of staff and the number of children within each ECEC facility. The reason for including studies using register‐based data is that we want the review to be as comprehensive as possible, and we expect that only a minority of studies will have had the resources to observe the actual adult/child ratio throughout the day within each setting.

#### 3.1.4 Types of outcome measures

The objective of the review is to explore the impact of adult/child ratio and group size on both process characteristics of quality of care as well as on child outcomes. The review aims to explore both developmental child outcomes as well as child well‐being.

We will extract the following outcomes provided they have been assessed with measures which have been validated on other samples than the intervention sample (researcher observations, caregiver or parental ratings).

Examples of process characteristics of quality: caregiver/child interaction, positive/negative affect, caregiver sensitivity, responsiveness, warmth, nurturing behaviour.

Examples of measures:

- The Early Childhood Environment Rating Scale (ECERS; Harms, Clifford, & Cryer, 1980; Vermeer, van Ijzendoorn, Cárcamo, & Harrison, 2016).
- The Infant/Toddler Environment Rating Scale (ITERS; Harms, Cryer, & Clifford,^1990^)
- The Arnett Caregiver Interaction Scale (CIS; Arnett, 1989). The Classroom Assessment Scoring System (Pianta, La Paro, & Hamre, 2008).

Examples of child outcomes: developmental data on language, motor, or interpersonal skills, child mental and physical health, behaviour problems, child well‐being, prosocial behaviour and psychological adjustment, pre‐math and pre‐literacy measures.

Examples of measures:

- The Strengths and Difficulties Questionnaire (SDQ; Goodman, 2001)
- The Child Behaviour Questionnaire (CBQ; Rutter, Tizard, & Whitmore, 1970)
- Preschool Measure of Attachment (Crittenden 1992)
- Infant and Toddler Social and Emotional Adjustment Scale (ITSEA; Carter & Briggs‐Gowan, 2000)
- Test of Preschool Early Literacy (TOPEL; Lonigan, Wagner, Torgesen, & Rashotte, 2007)
- Preschool Early Numeracy Skills Screener‐Brief or PENS‐B (Purpura, Reid, Eiland, & Baroody, 2015).
- The Woodcock‐Johnson Tests og Cognitive Ability (Woodcock, 1997).

Studies will be included if at least one reliable measure of adult/child ratio or group size and at least one of the outcomes mentioned above are reported.

Eligible outcome measures are not limited to the ones mentioned above

##### Primary outcomes

Based on the objectives of the present review, we do not distinguish between primary and secondary outcomes.

##### Secondary outcomes

###### Duration of follow‐up

Follow‐up at any given point in time will be included if meaningful based on the objectives for the review. This means that if possible, we will include follow‐up data regarding children's development and well‐being throughout the children's life course. If we include follow‐up data, we will examine if the effects differ across the length of follow‐up in the moderator analysis.

###### Types of settings

In this review we will examine the impact of adult/child ratio and group size in formal ECEC settings with children aged 0–5 years. Thus, we will exclude studies of informal care arrangements such as private babysitters or family members. Furthermore, we will exclude studies of children living in residential care arrangements such as foster families or institutions. The reasons for excluding studies of children living in residential care arrangements is that the objective of this review is to explore the impact of adult/child ratio and group size on child development and well‐being of children who are enroled in some form of formal nonparental ECEC during the day and not children being cared for around the clock by nonparental caregivers.

### 3.2 Search methods for identification of studies

#### 3.2.1 Search strategy

Relevant studies will be identified through searches in electronic databases, grey literature repositories & resources, hand searches in specific targeted journals, citation tracking, contact to international experts and internet search engines. Following bibliographic databases will be searched:

- SocINDEX
- PsycINFO
- EconLit
- ERIC
- Teacher Reference Center
- Academic Search
- Science Citation Index
- Social Science Citation Index
- Sociological Abstracts
- PubMed/MEDLINE

##### Electronic searches

An example of the search strategy used for the databases on the EBSCO‐host platform is listed as follows:

**Table:** 

Search	Search terms
S10	S8 OR S9
S9	S6 OR S7
S8	S4 AND S5
S7	TI (caretaker* OR teacher* OR staff* OR caregiver* OR adult*) AND TI ratio
S6	AB (caretaker* OR teacher* OR staff* OR caregiver* OR adult*) N8 ratio N8 (child* OR infant* OR toddler* OR “child care center*” OR "child care centre*" OR “child care home*”)
S5	TI (“group size*” OR class size*) OR AB (“group size*” OR class size*)
S4	S1 OR S2 OR S3
S3	TI care N2 (TI center* OR TI centre* OR TI day* OR TI child*) OR AB care N2 (AB center* OR AB centre* OR AB day* OR AB child*)
S2	TI (“Early childhood*” OR preschool* OR “non parental” OR kindergarten*) OR AB (“Early childhood*” OR preschool* OR “non parental” OR kindergarten*)
S1	TI (Infant* OR toddler* OR child* OR pupil* OR student* OR newborn* OR neonate* OR baby OR babies) AB (Infant* OR toddler* OR child* OR pupil* OR student* OR newborn* ORneonate* OR baby OR babies)

##### Searching other resources

The following grey literature resources will be searched:

- ProQuest Dissertations & Theses Global
- EBSCO Open Dissertations
- Open Grey
- Google Scholar
- Google searches
- Evidence Base (international repository for systematic reviews in the field of education)
- Campbell Library
- Cochrane Library
- Centre for Reviews and Dissemination Databases
- EPPI‐Centre Systematic Reviews – Database of Education Research
- Social Care Online
- Social Science Research Network

##### Hand search

A number of specific journals will be hand‐searched. We will decide upon which journals to hand search based on the identified records from the electronic searches. The following are examples of specific journals which we may decide to hand search:

- *Scandinavian Journal of Educational Research*
- *Nordic Studies in Education*
- *European Early Childhood Education Research Journal*
- *Early Child Development and Care*
- *Early Childhood Education Journal*
- *Journal of Early Childhood Research*
- *International Journal of Early Childhood*
- *International Research in Early Childhood Education*
- *Contemporary Issues in Early Childhood*
- *Journal of Early Childhood Teacher Education*
- *Child Care in Practice*
- *Childhood*
- *American Educational Research Journal*
- *Learning Environments Research*
- *Child Development*
- *Developmental Psychology*
- *Early Childhood Research Quarterly*
- *Early Education and Development*

##### Citation tracking

In order to identify both published studies and grey literature we will utilize citation‐tracking/snowballing strategies. Our primary strategy will be to citation‐track related systematic‐reviews and meta‐analyses. The review team will also check reference lists of included primary studies for new leads.

##### Contact with international experts

We will contact international experts to identify unpublished and ongoing studies.

##### Citation tracking

In order to identify both published studies and grey literature we will utilize citation‐tracking/snowballing strategies. Our primary strategy will be to citation‐track related systematic‐reviews and meta‐analyses. The review team will also check reference lists of included primary studies for new leads.

##### Contact with international experts

We will contact international experts to identify unpublished and ongoing studies.

### 3.3 Data collection and analysis

#### 3.3.1 Description of methods used in primary research

We will include three main types of study designs in this review: RCT, QRCT, and QES comparing different caregiving settings. In addition, studies using a repeated‐measures experimental design in which the same caregiver and/or children are observed under different conditions within a short time span will also be included. Since the aim of the study is to explore the causal impact of adult/child ratio and group size, studies reporting associations in cohort, cross‐sectional or longitudinal designs will not be included, unless they include a relevant comparison group.

With regards to the anticipated methods encountered in the included studies, we expect that a significant amount of studies will be conducted without randomization of participants. The reason for including studies without full randomization of participants is that we wish for the review to be as comprehensive as possible. Excluding nonrandomized studies would carry the risk of losing vital information of relevance to the review question.

An example of a study that may be included in the review is that of Russell (1990) which investigated the effects of small changes in child‐staff ratios on child and staff behaviour in 27 preschools. In this study, the numbers of children were manipulated to create a “low” ratio, an “average” ratio and a “high” ratio. The results of the study pointed to a greater ratio effect on individual child behaviour than on individual staff behaviour. With regards to whole group behaviour, staff members had to deal with substantial increases in problematic child behaviours under lower ratios, just as child access to staff on an individual or small group basis was reduced.

In addition, Smith, McMillan, Kennedy, and Ratcliffe (1989) examined the effect of improving staff ratios in New Zealand kindergartens on the interactions between children and staff. The design included comparisons between four kindergartens who acquired additional staff and four contrast kindergartens who maintained their usual staffing. Results showed that the introduction of additional staff reduced children's negative peer behaviour. Furthermore, while staff behaviour showed fewer changes than child behaviour, kindergartens with additional staffing saw adults making more non‐verbal initiations to children, talking more to parents, involving themselves more in children's play and talking to other staff more. Authors concluded that additional staffing improved preschool quality, but confounding factors associated with “experiments in nature” (e.g., subject attrition) prevented more definitive findings.

#### 3.3.2 Criteria for determination of independent findings

##### Selection of studies

Under the supervision of review authors, two review team assistants will first independently screen titles and abstracts to exclude studies that are clearly irrelevant. Studies considered eligible by at least one assistant or studies where there is insufficient information in the title and abstract to judge eligibility will be retrieved in full text. The full texts will then be screened independently by two review team assistants under the supervision of the review authors. Any disagreement of eligibility will be resolved by the review authors. Exclusion of studies that otherwise might be expected to be eligible will be documented and presented in an appendix.

The study inclusion criteria will be piloted by the review authors (see Appendix A). The overall search and screening process will be illustrated in a flow diagram. None of the review authors will be blind to the authors, institutions, or the journals responsible for the publication of the articles.

##### Data extraction and management

Two review authors will independently code and extract data from included studies. A coding sheet will be piloted on several studies and revised as necessary (see Appendix A). Disagreements will be resolved by consulting a third review author with extensive content and methods expertise. Disagreements resolved by a third reviewer will be reported. Data and information will be extracted on available characteristics of participants, intervention characteristics and control conditions, research design, sample size, risk of bias and potential confounding factors, outcomes, and results. Extracted data will be stored electronically.

##### Assessment of risk of bias in included studies

We will assess the risk of bias in randomized studies using Cochrane's revised risk of bias tool, ROB 2 (Higgins, Savovic, Page, & Sterne, 2019).

The tool is structured into five domains, each with a set of signalling questions to be answered for a specific outcome. The five domains cover all types of bias that can affect the results of randomized trials.

The five domains for individually randomized trials are:

(1) bias arising from the randomization process;
(2) bias due to deviations from intended interventions (separate signalling questions for effect of assignment and adhering to intervention);
(3) bias due to missing outcome data;
(4) bias in measurement of the outcome;
(5) bias in selection of the reported results.

If we include cluster‐randomized trials, an additional domain is included ((1b) Bias arising from identification or recruitment of individual participants within clusters). We will use the latest template for completion (currently it is the version of 15 March 2019 for individually randomized parallel‐group trials and 20 October 2016 for cluster‐randomized parallel‐group trials).

We will assess the risk of bias in nonrandomized studies using the model ROBINS‐I, developed by members of the Cochrane Bias Methods Group and the Cochrane Non‐Randomised Studies Methods Group (Sterne, Hernán, et al., 2016). We will use the latest template for completion (currently it is the version of 19 September 2016).

The ROBINS‐I tool is based on the Cochrane RoB tool for randomized trials, which was launched in 2008 and modified in 2011 (Higgins et al., 2011).

The ROBINS‐I tool covers seven domains (each with a set of signalling questions to be answered for a specific outcome) through which bias might be introduced into nonrandomized studies:

(1) bias due to confounding;
(2) bias in selection of participants;
(3) bias in classification of interventions;
(4) bias due to deviations from intended interventions;
(5) bias due to missing outcome data;
(6) bias in measurement of the outcome;
(7) bias in selection of the reported results.

The first two domains address issues before the start of the interventions and the third domain addresses classification of the interventions themselves. The last four domains address issues after the start of interventions and there is substantial overlap for these four domains between bias in randomized studies and bias in nonrandomized studies (although signalling questions are somewhat different in several places, see Higgins et al., 2019; Sterne, Higgins, Elbers, Reeves, & The Development Group for ROBINS‐I, 2016).

Randomized study outcomes are rated on a “Low/Some concerns/High” scale on each domain, whereas nonrandomized study outcomes are rated on a “Low/Moderate/Serious/Critical/No Information” scale on each domain. The level “Critical” means that the study (outcome) is too problematic in this domain to provide any useful evidence on the effects of the intervention and it is excluded from the data synthesis. The same critical level of risk of bias (excluding the result from the data synthesis) is not directly present in the RoB 2 tool, according to the guidance to the tool (Higgins et al., 2019).

We will add a critical level of risk of bias to the RoB 2 tool with the same meaning as in the ROBINS‐I tool; that is, the study (outcome) is too problematic in this domain to provide any useful evidence on the effects of the intervention and it is excluded from the data synthesis. We will stop the assessment of a randomized study outcome using the RoB 2 as soon as one domain is judged as “Critical”. Likewise, we will stop the assessment of a nonrandomized study outcome as soon as one domain in the ROBINS‐I is judged as “Critical”.

“High” risk of bias in multiple domains in the RoB 2 assessment tool may lead to a decision of an overall judgement of “Critical” risk of bias for that outcome and it will be excluded from the data synthesis. “Serious” risk of bias in multiple domains in the ROBINS‐I assessment tool may lead to a decision of an overall judgement of “Critical” risk of bias for that outcome and it will be excluded from the data synthesis.

###### Confounding

An important part of the risk of bias assessment of nonrandomized studies is consideration of how the studies deal with confounding factors. Systematic baseline differences between groups can compromise comparability between groups. Baseline differences can be observable (e.g., age and gender) and unobservable (to the researcher; e.g., children's motivation and “ability”). There is no single nonrandomized study design that always solves the selection problem. Different designs represent different approaches to dealing with selection problems under different assumptions, and consequently require different types of data. There can be particularly great variations in how different designs deal with selection on unobservables. The “adequate” method depends on the model generating participation, that is, assumptions about the nature of the process by which participants are selected into a programme.

A major difficulty in estimating causal effects of adult/child ratio and group size is the potential heterogeneity of both the different ECEC settings and of the children. In addition to the prespecified confounding factors, there may be unobservable factors affecting child development and well‐being or invisible selection mechanisms causing certain types of families to choose a specific ECEC setting for their child for reasons unavailable to the researcher.

As there is no universally correct way to construct counterfactuals for nonrandomized designs, we will look for evidence that identification is achieved, and that the authors of the primary studies justify their choice of method in a convincing manner by discussing the assumption(s) leading to identification (the assumption(s) that make it possible to identify the counterfactual). Preferably the authors should make an effort to justify their choice of method and convince the reader that the children and settings with high versus low adult/child ratios and small versus large group sizes are comparable.

In addition to unobservables, we have identified the following observable confounding factors to be most relevant: age/gender of the child, special needs status, structural characteristics of the ECEC setting (such as preschool, private or centre‐based care, educational level of teachers/caretakers) and socioeconomic background and ethnicity of the families (minority status or not). In each study, we will assess whether these factors have been considered, and in addition we will assess other factors likely to be a source of confounding within the individual included studies.

###### Importance of prespecified confounding factors

The motivation for focusing on age/gender of the child, special needs status, structural characteristics of the ECEC setting (such as preschool, private or centre‐based care, educational level of teachers/caretakers) and socioeconomic background and ethnicity of the families (minority status or not) is given below.

The younger the child, the more dependent the child is on stimulating adult/child interaction and basic nurture (Howes et al. 1992). Therefore, the impact of adult/child ratio and group size may vary depending on the age of the children, with younger children benefiting more from higher ratios and smaller group sizes than older children.

From a very early age, gender is associated with differences in child behaviour and cognition (Chaplin & Aldao, 2013; Silverman, 2003; Ostrov & Keating, 2004). Little girls and boys often show different toy and play preferences (Todd, Barry, & Thommessen, 2017) and thus it is possible that gender may have an impact on what constitutes the best ECEC setting for each child.

Children with special needs such as physical or psychological disabilities are by definition considered to require more adult stimulation and care than children without any identified special needs and thus they may benefit more from an increased adult/child ratio and smaller group sizes.

In previous research, other structural aspects of the ECEC settings have been found to be associated with both process quality and child outcomes and thus we consider the nature of the care setting (private vs. centre‐based day care or preschool) as well as the educational level and continuous professional development of the teachers/caretakers to be potentially important confounders.

A large body of research documents the impact of parental socioeconomic background on almost all aspects of children's development (Renninger, Sigel, Damon & Lerner, 2006), which is why we consider it important to control for this.

For children aged 0–5 years, language acquisition is one of the most essential developmental tasks. Many ethnic minority children grow up to become bilingual and this may require more adult stimulation and interaction within ECEC settings. Thus, the potential impact of adult/child ratio and group size may vary depending on whether the child is monolingual or bilingual.

Children are often enroled in ECEC settings throughout the year based on their date of birth and not at a common point in time such as the beginning of the school year which would make the collection of true pre‐test scores (meaning pre‐enrolment scores) difficult. Therefore, we do not include pre‐test scores as a prespecified confounding factor. However, if pre‐test scores are available, these will be taken into account when we evaluate the credibility of the between‐group comparability.

###### Assessment

At least two review authors will independently assess the risk of bias for each relevant outcome from the included studies. Any disagreements will be resolved by a third reviewer with content and statistical expertise and will be reported. We will report the risk of bias assessment in risk of bias tables for each included study outcome in the completed review.

#### 3.3.3 Measures of treatment effect

##### Measures of effect

###### Continuous outcomes

For continuous outcomes, effect sizes with 95% confidence intervals will be calculated, where means, adjusted means/regression coefficients, and standard deviations are available. If means and standard deviations are not available, we will calculate standardized mean differences (SMDs) from *F*‐ratios, *t*‐values, *χ* ^2^ values and correlation coefficients where available, using the methods suggested by Wilson and Lipsey (2001). If insufficient information is yielded, the review authors will request this information from the principal investigators. Hedges' *g* will be used for estimating SMDs. Hedges' *g* and its standard error are calculated as (Wilson & Lipsey, 2001, pp. 47–49)

(1) $$\;g=[1–3/(4N-9)]\times (\beta /s_{p}),$$

(2) $$\;SE_{g}={\left[(N/(n_{1}+n_{2}))+(g^{2}/2N)\right]}^{0.5},$$

Where *N* = *n*_*1*_ + *n*_*2*_ is the total sample size, *β* is an estimate of the intervention effect (e.g., the postintervention difference in means between the intervention and control group), and *s*_p_ is the pooled standard deviation defined as

(3) $$\;s_{p}={\left[((n_{1}-1)s_{1}^{2}+(n_{2}-1)s_{2}^{2})/(n_{1}-1+n_{2}-1)\right]}^{0.5}.$$

Here, *s*_*1*_ and *s*_*2*_ denotes the raw standard deviation of the intervention and control group.

We will use covariate‐adjusted means or regression coefficients for the intervention effect estimates and the unadjusted post‐test standard deviation whenever available. Because we anticipate that many studies will not include the preintervention standard deviation, we will use the postintervention standard deviation.

We will use the same type of effect size measure for the single group repeated‐measures designs (as recommended by e.g., Morris & DeShon, 2002; Lakens, 2013). As the intervention group is its own control group in this design, standardization with the intervention and control group post‐test standard deviation is not feasible. We will instead calculate the effect size as (denoted Hedges' *g*_av_ in Lakens, 2013)

(4) $$g_{av}=[1–3/(4N-9)]\times (M_{diff}/[(sd_{1}+sd_{2})/2]),$$

where *M*_diff_ is the mean difference between an outcome measured at pre‐ and post‐test, *sd*_1_ is the standard deviation at pre‐test, and *sd*_2_ is the standard deviation at post‐test. We will calculate the standard error as for *g*. Another option would be to use *g*_rm_ (Morris & DeShon, 2002; Lakens, 2013), however, this effect size measure requires knowledge of the correlation between pre‐ and post‐test measures, which may not be available in our case.

We discuss how and when we will combine effect sizes from different research designs in Section 3.3.8 and how we test if our results are sensitive to combining effect sizes from different designs in Section 3.3.10.

###### Dichotomous outcomes

For dichotomous outcomes, we will calculate odds ratios with 95% confidence intervals. Attachment status (secure vs. insecure) and children with or without behaviour problems are examples of relevant dichotomous outcomes in this review. Should we find a large enough number of studies using dichotomous outcomes, we will test whether our results are sensitive to combining dichotomous and continuous outcome measures. If this is the case, we will also perform a sensitivity analysis using only dichotomous measures and the following procedure to calculate effect sizes: We will use the natural logarithm of odds ratios (LOR) in the calculations, together with 95% confidence intervals and *p*‐values, and then convert the results back to the original odds ratios once the meta‐analysis is performed. The LOR and its approximate standard error are calculated as (Wilson & Lipsey, 2001, pp. 53–54)

(5) LOR=log[(ad)/(bc)],

(6) $$SE_{LOR}={\left(1/a+1/b+1/c+1/d\right)}^{0.5},$$

where *a* is the frequency of “good” outcomes in the treatment group (e.g., the frequency of children with no behaviour problems), *b* is the frequency of “bad” outcomes in the treatment group (the frequency of children with behaviour problems), and *c* and *d* are the frequencies of good and bad outcomes in the control group, respectively.

#### 3.3.4 Unit of analysis issues

We will take into account the unit of analysis of the studies to determine whether individuals were randomized in groups (i.e., cluster‐randomized trials), whether individuals may have undergone multiple interventions, whether there were multiple treatment groups and whether several studies are based on the same data source.

##### Cluster‐randomized trials

The randomization of clusters can result in an overestimation of the precision of the results (with a higher risk of a Type I error) where their use has not been compensated for in the analysis. If we include cluster RCTs, the impact of the inclusion of data from such studies in the meta‐analyses will be explored using a sensitivity analysis and any necessary adjustments to the data will be made using available estimates of ICC and the methods described in Hedges (2007).

##### Multiple intervention groups and multiple interventions per individual

Studies with multiple intervention groups with different individuals, and studies using multiple tests for the same intervention groups, will be included in the review. To avoid problems with dependence between effect sizes, we will use the robust variance estimation (RVE) methods developed by Hedges, Tipton, and Johnson (2010). We will use the results in Tanner‐Smith and Tipton (2014) and Tipton (2015) to evaluate if there are enough studies for this method to consistently estimate the standard errors. That is, we will report if the adjusted degrees of freedom are close to or below 4, as the results in Tanner‐Smith and Tipton (2014) and Tipton (2015) indicate that the standard errors are not reliable below this level.

##### Multiple studies using the same sample of data

In some cases, several studies may have used the same sample of data or some studies may have used only a subset of a sample used in another study. We will review all such studies, but in the meta‐analysis we will only include one estimate of the effect for each outcome from each sample of data. This means that if the same outcome is reported for a subgroup and for the full sample in separate studies, we will only include the study using the full set of participants.

#### 3.3.5 Dealing with missing data

Missing data in the individual studies will be assessed using the risk of bias tool. Studies must permit calculation of a numeric effect size for the outcomes to be eligible for inclusion in the meta‐analysis. Where studies have missing summary data, such as missing standard deviations, we will derive these where possible from, for example, *F*‐ratios, *t*‐values, *χ* ^2^ values and correlation coefficients using the methods suggested by Wilson and Lipsey (2001). If these statistics are also missing, the review authors will request information from the study investigators.

If missing summary data necessary for the calculation of effect sizes cannot be derived or retrieved, the study results will be reported in as much detail as possible, that is, the study will be included in the review but excluded from the meta‐analysis.

#### 3.3.6 Assessment of heterogeneity

We will investigate the following factors with the aim of explaining potential observed heterogeneity: study‐level summaries of participant characteristics (e.g., studies considering a specific population such as at‐risk children, age group or studies where separate effects for low/high socioeconomic status are available).

#### 3.3.7 Assessment of reporting biases

Reporting bias refers to both publication bias and selective reporting of outcome data and results. Here, we state how we will assess publication bias.

We will use funnel plots for information about possible publication bias if we find sufficient studies (Higgins & Green, 2011). However, asymmetric funnel plots are not necessarily caused by publication bias (and publication bias does not necessarily cause asymmetry in a funnel plot). If asymmetry is present, we will consider possible reasons for this.

#### 3.3.8 Data synthesis

The overall data synthesis will be conducted where effect sizes can be calculated. We hope to be able to perform multiple random‐effects meta‐analyses based on SMDs (Hedges' *g*) and use the RVE procedure developed by Hedges et al. (2010). In addition to the advantage that we can include all relevant effect sizes in the analysis the procedure calculates standard errors using an empirical estimate of the variance: it does not require any assumptions regarding the distribution of the effect size estimates. We will use the *robumeta* package in R (Fisher, Tipton, & Zhipeng, 2017) and the correlated effects weighting scheme to implement the RVE procedure. This weighting scheme uses estimates of the between and within‐study variance, and an initial value of the within‐study effect size correlation (*ρ*) to calculate the weights used in the random‐effects analysis. We will use the default value of *ρ* = 0.80 and conduct sensitivity tests with a variety of values to asses if the general results are robust to the choice of *ρ*. We will use the small sample adjustment to the residuals used in RVE and the Satterthwaite degrees of freedom for significance tests (Tipton, 2015), reporting 95% confidence intervals throughout.

The results in Tipton (2015) suggest that the degrees of freedom depend not only the number of studies but also on the type of covariates included in the meta‐regression. The degrees of freedom can be few, even when the number of studies is large, and if a covariate is unbalanced or a covariate with high leverage is included, the degrees of freedom will vary from coefficient to coefficient. The corrections to the degrees of freedom enable us to assess when the RVE procedure performs well. As suggested by Tanner‐Smith and Tipton (2014) and Tipton (2015), if the degrees of freedom are fewer than four, the RVE results should not be trusted.

If we include data with binary outcomes such as children with and without behaviour problems or children with or without mental health symptom scores above the clinical cut‐off for a given measure, we will calculate odds ratios as outlined in Section *Measures of effect*. There are statistical approaches available to re‐express dichotomous and continuous data so that they can be pooled (Sánchez‐Meca, Marín‐Martínes & Chacón‐Moscoso, 2003). In order to calculate a common metric, odds ratios will be converted to SMDs using the Cox transformation. We will only transform dichotomous effect sizes to SMD's if appropriate, as may be the case with the outcomes "attachment" and "behaviour problems" that can be measured with binary and continuous data.

If we include studies using different metrics, we will conduct a sensitivity analysis to compare the meta‐analytic results with and without the converted studies. When effect sizes cannot be pooled, study‐level effects will be reported in as much detail as possible.

As different estimation methods may produce effect sizes that are not comparable, we will be transparent about all methods used in the primary studies (research design and statistical analysis strategies) and use caution when synthesizing effect sizes. For example, in single group repeated‐measures designs, children and caregivers act as their own control group. As the standard deviation is therefore based on a more homogeneous group of children/caregivers than in intervention‐control group designs, there is a risk that the standard deviations are smaller in single group repeated‐measures designs. Consequently, effect sizes risk being inflated compared with intervention‐control group designs (i.e., the same absolute effect will mechanically result in a larger effect size, if the standard deviation is smaller). However, if for example, time‐varying contextual factors have a strong influence on a measure, then there may instead be more variation in single group repeated‐measures designs. Although the latter situation seems less likely in our case, it is difficult to rule out completely beforehand as is the possibility that the standard deviations are approximately equal. We will, therefore, include effect sizes from single group repeated‐measures designs in our primary analysis. We describe how we will test the sensitivity to the inclusion of effect sizes using different research designs and statistical methods, including single group repeated‐measures designs, in Section 3.3.10.

In our primary analysis, we will estimate the effects separately by conceptual outcome and intervention type. By conceptual outcome, we mean that we may choose to combine different measures if they measure the same or very similar underlying phenomena, such as children's mental health, caregiver‐child interaction, or language skills. As discussed in Section 1.2, included interventions are of three types: (a) interventions that only change the adult/child ratio (e.g., that employ an extra preschool teacher for an existing group of children), (b) interventions that only change the group size (e.g., that split one group of two teachers and ten children into two groups of one teacher and five children), and (c) interventions that change both the adult/child ratio and the group size (e.g., when a group with one teacher is increased from five to six children).

As changes to both adult/child ratios and group sizes can be small and large, and effects may differ depending on the baseline ratio/size, we would ideally want to estimate separate effects for the different intervention types and categories defined by the size of the change and the baseline. We believe that this type of estimation strategy would come closest to answering the question of what the optimal adult/child ratio and group size are and it would make relatively weak assumptions about for example the functional form of the relation between effect sizes, and adult/child ratios and group sizes. However, as previous reviews (Perlman et al., 2017) found few studies, it seems unlikely that we will find enough studies for this estimation strategy to be feasible.

If this strategy is not feasible, we will estimate a weighted average effect for each intervention type by specifying regressions with *g* as the outcome variable and a single indicator (i.e., just an intercept) for each type of intervention, as the explanatory variable. We will code the indicator so that it represents improvements, that is, increased adult/child ratios and decreased group sizes. Note that it is conceptually possible, but perhaps unlikely, that an intervention may increase the adult/child ratio (an improvement) and simultaneously increase the group size (a deterioration) or vice versa. If we include such interventions, we will include two indicators, one for interventions where both the ratio and group size improve and one for “mixed” interventions, in the regressions for intervention type 3.

The coefficient on the indicator in these regressions gives us an estimate of the weighted average effect size in the categories defined by conceptual outcome and intervention type. This strategy also makes relatively weak assumptions about the functional form of the relation between effect sizes and ratios and group sizes. As preschool decision‐makers may want to choose between changing adult/child ratios, group sizes, or both, obtaining a separate estimate for the three intervention types is policy relevant. However, the estimation strategy may mix large and small changes from different baselines, and it may not make optimal use of the available information. In the moderator analysis, described next, we will, therefore, try a different strategy, which include adult/ratios and group sizes as continuous variables and collapses the three intervention types.

There may also be important differences between interventions regarding, for example, the ages of children, duration of the interventions (see e.g., de Schipper et al., 2006; Smith et al. 1989), and the measurement timing. However, we expect that most studies measure effects close to the end of intervention for comparable ages and that the duration of the intervention also influences the outcome measures chosen (e.g., measuring the development of language skills is not meaningful if the intervention is very short, as in de Schipper et al., 2006). As it is also difficult to define cutoffs for these variables that are not arbitrary, we will test whether effect sizes differ across these variables in the moderator analysis rather than estimate separate regressions for pre‐defined categories.

#### 3.3.9 Subgroup analysis and investigation of heterogeneity

If the number of included studies is sufficient and there is variation in the covariates, we will perform moderator analyses to explore how observed variables are related to heterogeneity. We will apply the RVE procedure, but, as indicated above, use a different strategy that we believe will increase statistical power and therefore allow us to examine more of the potentially important moderators, as well as examine how the size of the change of adult/child ratios and group sizes are associated with effect sizes. The price of these advantages comes primarily in the form of making stronger functional form assumptions. As the moderator analysis is exploratory rather than confirmatory (Thompson & Higgins, 2002), for example, because it includes study‐level variables that were not (quasi‐)experimentally manipulated in interventions, we believe this trade‐off is acceptable.

We will keep estimating separate regressions for conceptual outcomes but collapse the three intervention types and include continuous variables measuring the changes to adult/child ratios and group sizes. To reduce the problem that the effect of the same incremental change to group size may be very different depending on the baseline group size, we will express the changes in percent (using the control group/pre‐test group size as the baseline rate). That is, a change from 3 to 4 children will not be same as a change from 33 to 34, as the first change is equal to a (4 − 3)/3 = 33.3% increase and the second a (34 − 33)/33 = 3.0% increase. Adult/child ratios will also be expressed as percent changes (e.g., in the example, changing from 1/3 to 1/4, again using the control group/pre‐test ratio as the baseline, amount to a decrease of (1/4 − 1/3)/(1/3) = −25.0%). That is, we specify the following type of regression equation:

(7) $$g_{ios}=\beta_{1}\Delta AC_{ios}+\beta_{2}\Delta GS_{ios}+e_{ios},$$

where *g*_*ios*_ is effect size *i* measured by conceptual outcome *o* from study *s*, Δ*AC*_*ios*_ is the change of the adult/child ratio in percent for this effect size, Δ*GS*_*ios*_ is the change in the group size in percent, *β*_*1*_ and *β*_*2*_ are parameters to be estimated, and *e*_*ios*_ is an error term (clustered by study in the RVE procedure). Expressing the changes in percent still entails a strong assumption that the relations between effect sizes and percent changes in group sizes and ratios are linear. However, this assumption seems at least less strong than the assumption that the relation would be linear in the absolute changes.

To this specification, we will add the following moderators (conditional on data being available and that there is variation in the data): mean age of the children receiving an intervention, intervention duration, length of follow‐up, the proportion of low SES children, an indicator for the type of pedagogy used in the country or region (separating between “early‐education” and “comprehensive/social pedagogy” approaches, see Wall, Litjens, & Taguma, 2015), and an indicator for interventions in the OECD countries. As moderators may be correlated, we prefer to include all variables in one regression. However, adding all moderators simultaneously may not be feasible, as it decreases the degrees of freedom. If this is the case, we prioritize moderators in the order mentioned above. That is, we will first add mean age, then intervention duration, length of follow‐up, the proportion of low SES, the pedagogy indicator, and lastly the OECD indicator, stopping when we risk not being able to reliably estimate a previously added variable (i.e., when the adjusted degrees of freedom <4).

We will report 95% confidence intervals for all moderator analyses. Conclusions from meta‐regression analyses will be cautiously drawn and will not solely be based on significance tests. The magnitude of the coefficients and width of the confidence intervals will be taken into account as well.

#### 3.3.10 Sensitivity analysis

Sensitivity analysis will be carried out by restricting the meta‐analysis to a subset of all studies included in the original meta‐analysis and will be used to evaluate whether the pooled effect sizes are robust across components of risk of bias. We will consider sensitivity analysis for each domain of the risk of bias checklists and restrict the analysis to studies with a low risk of bias.

Sensitivity analyses with regard to research design and statistical analysis strategies in the primary studies to ensure that different methods produce consistent results. We will estimate separate regressions for different research designs (e.g., intervention‐control group designs and single group repeated‐measures designs) and statistical methods (e.g., estimating effects by comparing raw means or by covariate‐adjusted regression coefficients) and add indicators of research designs and methods in the regressions used in the moderator analysis.

As mentioned in Section 3.3.8, we will re‐estimate our primary analysis using different values of *ρ*, and estimate separate effects for different effect size measures (e.g., originally dichotomous effect sizes and SMDs).

##### Treatment of qualitative research

We do not plan to include qualitative research.

## AUTHOR CONTRIBUTIONS

Nina Thorup Dalgaard is a psychologist, PhD. Nina has previously worked as both an educational psychologist within a primary school setting and as a clinical child psychologist and thus has knowledge about the socioemotional and cognitive development of children.

Anja Bondebjerg holds a Master's degree in Sociology and has worked extensively with systematic reviews and research mappings in the fields of education and early childhood education and care. She is knowledgeable regarding the structure and process of conducting systematic reviews

Rasmus Klokker holds an MSc in sociology has worked on systematic reviews mapping research on daycare and preschool in the Nordic countries and has general knowledge on the field of sociology of education. Rasmus Klokker is thus knowledgeable on the scholarly literature concerning daycare and preschool, and has general knowledge on educational institutions within a sociological framework. Rasmus has worked on and assisted the completion of several systematic reviews within the Campbell framework. Rasmus Klokker has been involved in all facets of conducting systematic reviews, and has completed a course, lead by Michael Borenstein, on meta‐analysis.

## SYSTEMATIC REVIEW METHODS

Jens Dietrichson is an experienced systematic reviewer and methodologist, having completed a number of systematic reviews as well as primary studies in the fields of education and early childhood education and care. He is currently the lead reviewer on three ongoing Campbell Systematic Reviews and is knowledgeable regarding all major facets of meta‐analytic methods and their application.

Anja Bondenjerg (please see description above)

## STATISTICAL ANALYSIS

Jens Dietrichson (please see description above)

Rasmus Klokker (please see description above)

## INFORMATION RETRIEVAL

Bjørn Christian Arleth Viinholt (information specialist): has 4 years of experience in developing and writing systematic reviews. As a part of undertaking systematic reviews, Bjørn has experience in developing systematic search strategies and processes of reference management. Bjørn will contribute with assisting and development of the systematic search strategy, executing the searches, and assist with reference management and grey literature searches. Bjørn will also assist with aspects relating to systematic literature searches in Campbell review methodology.

## DECLARATIONS OF INTEREST

We do not have any potential conflicts of interest.

## Footnotes

^1^

https://www.oecd.org/els/soc/PF3_2_Enrolment_childcare_preschool.pdf.

^2^

https://www.boerneraadet.dk/media/30309/Miniboernepanel‐Mellem‐hjem‐og‐boernehave.pdf.

^3^

https://data.worldbank.org/indicator/SE.PRE.ENRR.

## APPENDIX A FIRST AND SECOND LEVEL SCREENING

First level screening is on the basis of titles and abstracts. Second level is on the basis of full text.

Reference id. No.:

Reviewers initials:

Source:

Year of publication:

Country/countries of origin:

Author(s):

The study will be excluded if one or more of the answers to Questions 1–4 are “No”. If the answers to Questions 1 to 4 are “Yes” or “Uncertain”, then the full text of the study will be retrieved for second level eligibility. All unanswered questions need to be posed again on the basis of the full text. If not enough information is available, or if the study is unclear, the author of the study will be contacted if possible.

**Screening questions:**

1. Does the study measure adult/child ratio and/or group size in early childhood education or care setting(s)?
  Yes ‐ include
  No – if no then stop here and exclude
  Uncertain ‐ include
  Question 1 guidance:
  The population of this review are children aged 0–5 years. Studies focusing on adult/child ratio or group size in educational settings with older children will not be eligible.
2. Do the study outcomes involve measures of process characteristics of the quality of care and/or child outcomes?
  Yes ‐ include
  No – if no then stop here and exclude
  Uncertain – include
  Question 2 guidance:
  The objective of the review is to explore the impact of adult/child ratio and group size on both *process characteristics of quality of care* as well as on *child outcomes*.
  Examples of process characteristics of quality: caregiver/child interaction, positive/negative affect, caregiver sensitivity, responsiveness, warmth, nurturing behaviour.
  Examples of child outcomes: developmental data on language, motor, or interpersonal skills, child mental and physical health, child behaviour problems, child well‐being, prosocial behaviour, pre‐math and pre‐literacy measures. Studies focusing on outcomes such as teacher/caretaker sickness or absenteeism will not be eligible.
3. Is the report/article a quantitative study with a comparison condition??

Yes ‐ include

No – if no then stop here and exclude

Uncertain – include

Question 4 guidance:

We are only interested in primary quantitative studies with a comparison group. Eligible study designs are: randomized controlled trials (RCTs), Quasi‐randomized controlled trial designs (QRCTs), Quasi‐experimental studies (QES) and repeated‐measures experimental designs in which the same caregiver and/or children are observed under different conditions within a short time span. Studies reporting associations in cohort, cross‐sectional and longitudinal study designs without a comparison group are not eligible.

We are not interested in theoretical papers on the topic or surveys/reviews of studies of the topic. (This question may be difficult to answer on the base of titles and abstracts alone.).

### Data extraction

**Table:**

**Names of author(s)**
**Title**
**Language**
**Journal**
**Year**
**Country**
**Type of ECEC setting (home based, centre based or preschool)**
**Participant characteristic (children's age range)**
**Programme feature:** *Study design*, (brief description)
**Programme feature:** *Intervention* (adult/child ratio and/or group size)
**Programme feature** *Outcomes* **:**
**Programme feature** *Participants, (At risk, minority, special needs etc)*
**Programme feature** *teacher/caretaker characteristics,* (educational background, years of experience, continuous professional development)
**Type of data used in study (independent observation, questionnaire, other (specify))**
**Level of aggregation (individual and/or setting)**
**Time period covered by analysis (divide into intervention and follow up)**
**Sample size (divide into treated/comparison)**

**Outcome measures**

Instructions: Please enter outcome measures in the order in which they are described in the report. Note that a single outcome measure can be completed by multiple sources and at multiple points in time (data from specific sources and time‐points will be entered later).

*Repeat as needed

**OUT COME DATA**

**DICHOTOMOUS OUTCOME DATA**

Repeat as needed

**CONTINUOUS OUTCOME DATA**

*Repeat as need

### Assessment of risk of bias in included studies

***User guide for unobservables***

Systematic baseline differences between groups can compromise comparability between groups. Baseline differences can be observable (e.g., age and gender) and unobservable (to the researcher; e.g., motivation and “ability”). There is no single nonrandomized study design that always solves the selection problem. Different designs solve the selection problem under different assumptions and require different types of data. Especially how different designs deal with selection on unobservables varies. The “right” method depends on the model generating participation, that is, assumptions about the nature of the process by which participants are selected into a programme.

As there is no universal correct way to construct counterfactuals we will assess the extent to which the identifying assumptions (the assumption that makes it possible to identify the counterfactual) are explained and discussed (preferably the authors should make an effort to justify their choice of method). We will look for evidence that authors using e.g. (this is NOT an exhaustive list):

**Natural experiments:**

Discuss whether they face a truly random allocation of participants and that there is no change of behaviour in anticipation of e.g. policy rules.

**Matching (including propensity scores):**

Explain and discuss the assumption that there is no selection on unobservables, only selection on observables.

**(Multivariate, multiple) Regression:**

Explain and discuss the assumption that there is no selection on unobservables, only selection on observables. Further discuss the extent to which they compare comparable people.

**Regression Discontinuity (RD):**

Explain and discuss the assumption that there is a (strict!) RD treatment rule. It must not be changeable by the agent in an effort to obtain or avoid treatment. Continuity in the expected impact at the discontinuity is required.

**Difference‐in‐difference (Treatment‐control‐before‐after):**

Explain and discuss the assumption that the trends in treatment and control groups would have been parallel, had the treatment not occurred.
